# Locus-Specific Isolation of the *Nanog* Chromatin Identifies Regulators Relevant to Pluripotency of Mouse Embryonic Stem Cells and Reprogramming of Somatic Cells

**DOI:** 10.3390/ijms232315242

**Published:** 2022-12-03

**Authors:** Arun Kumar Burramsetty, Ken Nishimura, Takumi Kishimoto, Muhammad Hamzah, Akihiro Kuno, Aya Fukuda, Koji Hisatake

**Affiliations:** 1 Laboratory of Gene Regulation, Faculty of Medicine, University of Tsukuba, 1-1-1 Tennodai, Tsukuba 305-8575, Japan; 2 Laboratory of Animal Resource Center, Department of Anatomy and Embryology, Faculty of Medicine, University of Tsukuba, 1-1-1 Tennodai, Tsukuba 305-8575, Japan; 3 Ph.D. Program in Human Biology, School of Integrative and Global Majors, University of Tsukuba, Tsukuba 305-8575, Japan

**Keywords:** dCas9, CAPTURE, *Nanog*, pluripotency, reprogramming

## Abstract

Pluripotency is a crucial feature of pluripotent stem cells, which are regulated by the core pluripotency network consisting of key transcription factors and signaling molecules. However, relatively less is known about the molecular mechanisms that modify the core pluripotency network. Here we used the CAPTURE (CRISPR Affinity Purification in situ of Regulatory Elements) to unbiasedly isolate proteins assembled on the *Nanog* promoter in mouse embryonic stem cells (mESCs), and then tested their functional relevance to the maintenance of mESCs and reprogramming of somatic cells. Gene ontology analysis revealed that the identified proteins, including many RNA-binding proteins (RBPs), are enriched in RNA-related functions and gene expression. ChIP-qPCR experiments confirmed that BCLAF1, FUBP1, MSH6, PARK7, PSIP1, and THRAP3 occupy the *Nanog* promoter region in mESCs. Knockdown experiments of these factors show that they play varying roles in self-renewal, pluripotency gene expression, and differentiation of mESCs as well as in the reprogramming of somatic cells. Our results show the utility of unbiased identification of chromatin-associated proteins on a pluripotency gene in mESCs and reveal the functional relevance of RBPs in ESC differentiation and somatic cell reprogramming.

## 1. Introduction

Pluripotent stem cells (PSCs) such as embryonic stem cells (ESCs) and induced pluripotent stem cells (iPSCs) have a remarkable ability to give rise to all types of cells in the body and have great potential in applications for cell-based regenerative medicine. PSCs can self-renew indefinitely and differentiate to form all three germ layers in response to appropriate cues [1,2,3]. The identity of PSCs is regulated by extracellular signals as well as the autoregulatory pluripotency network that includes transcription factors such as OCT4, SOX2, and NANOG [4,5]. The importance of the core pluripotency transcription factors is underscored by their requirement or utility for reprograming of somatic cells to iPSCs [6]. To maintain the pluripotent state, PSCs have a relatively open and highly dynamic chromatin [7], consisting of decondensed chromatin with less abundant heterochromatin, which allows pervasive transcription [8,9].

The autoregulatory pluripotency network should be dismantled when ESCs differentiate to form the three germ layers. Upon differentiation of ESCs, dramatic changes occur in genome-wide binding patterns of transcription factors and the chromatin landscape including histone modifications and DNA methylation [10,11,12], which are accompanied by massive reorganization in the higher-order chromatin architecture [13]. However, outside of the core pluripotency network and its associated chromatin regulators, the molecular switches that change the ESC program from self-renewal to differentiation remain to be more fully explored. Such molecular switches are presumably associated with the core pluripotency network; therefore, one feasible strategy to explore these molecules would be the locus-specific isolation of the native chromatin from ESCs.

In recent years, several innovative methods have been developed to isolate proteins assembled on the native chromatin in cells. For example, PICh (Proteomics of Isolated Chromatin segments) utilizes a specific nucleic acid probe to isolate fragmented native chromatin from cells [14]. The use of nucleic acid hybridization, however, does not necessarily provide sufficient fold enrichment for isolating native chromatin from a single genomic locus. Another method termed insertional chromatin immunoprecipitation (iChIP) utilizes a LexA-recognition sequence inserted near the target locus, and immunoprecipitation of LexA enriches the native chromatin of the target locus [15]. A modified version of iChIP, termed engineered DNA binding molecule mediated chromatin immunoprecipitation (enChIP), utilizes epitope-tagged transcription activator-like effectors (TALEs) in place of LexA to precipitate the native chromatin [16]. However, inserting the LexA binding sequence into the target locus or generating TALEs that bind specifically to the target sequence is laborious and time-consuming.

More recent studies reported applications of CRISPR-based genome targeting to isolate the specific chromatin region from cells. CRISPR/dCas9-based enChIP enables targeting of the chromatin by using dCas9 and an appropriate gRNA in place of TALE or LexA [17]. The dCas9-APEX-mediated method utilizes dCas9 fused with peroxidase APEX2 [18]. In this method, tyrosine residues of chromatin proteins adjacent to the dCas9-APEX complex are biotin-labeled by the action of APEX1-oxidized biotin phenol. A more efficient method termed CAPTURE (CRISPR Affinity Purification in situ of Regulatory Elements) combines the specificity of biotin-streptavidin binding with the convenience of the CRISPR/dCas9 system to isolate the target chromatin region [19]. Because of the versatility of dCas9 binding and the high specificity of the biotin-streptavidin interaction, we chose CAPTURE to isolate the native chromatin of a pluripotency-associated gene *Nanog* from ESCs and identified proteins that function in ESC differentiation and iPSC generation.

## 2. Results

### 2.1. CAPTURE of the Nanog Promoter

**Figure 1:**
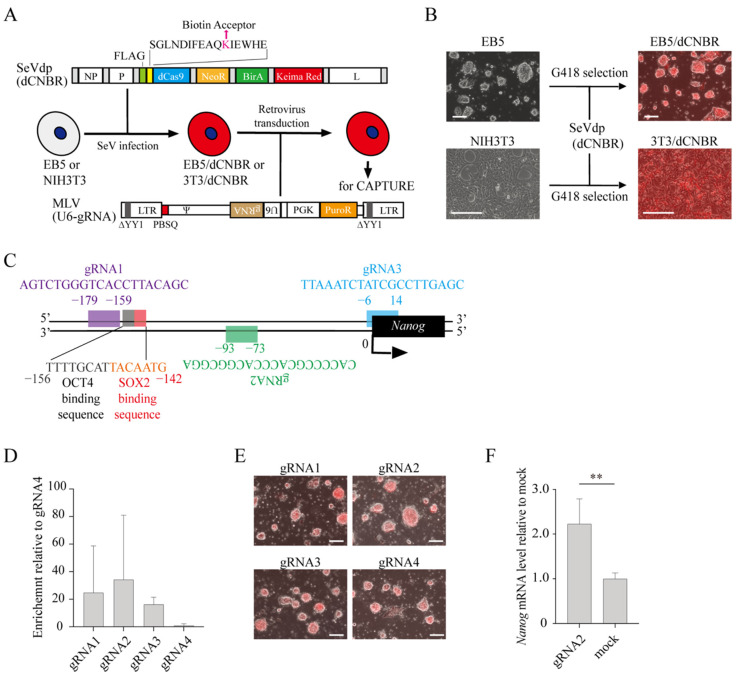
Generation of mouse embryonic stem cells (mESCs) for CAPTURE of the *Nanog* promoter. (**A**) Structure of vectors and experimental procedure for the CAPTURE method. SeVdp(dCNBR) vector expresses proteins for CAPTURE including dCas9, the neomycin resistant gene (NeoR), biotin ligase (BirA) and Keima-Red. dCas9 was fused with a FLAG-tag and biotin acceptor sequence at its N-terminus. After selection of SeVdp(dCNBR)-infected mESCs or NIH3T3 cells (EB5/dCNBR or 3T3/dCNBR, respectively), MLV(U6-gRNA) was transduced to express guide RNA (gRNA) as well as the puromycin resistant gene (PuroR). NP, P, and L; genes encoding Sendai virus NP, P, and L proteins, respectively. ∆YY1 and PBSQ; mutations in the YY1-binding site and primer-binding site, respectively. LTR; long terminal repeat. ψ, packaging signal. U6; U6 promoter. PGK; PGK promoter. (**B**) Keima-Red expression from SeVdp(dCNBR). EB5 and NIH3T3 cells were infected with SeVdp(dCNBR) followed by selection with 800 µg/mL and 1 mg/mL of G418 for 3 and 7 days, respectively. Scale bars, 200 µm. (**C**) Positions of gRNA target sequences on each strand of the *Nanog* promoter. OCT4 and SOX2 binding motifs are also indicated. (**D**) Enrichment of the *Nanog* promoter DNA by CAPTURE with gRNAs. Chromatins were collected from EB5/dCNBR cells expressing the indicated gRNA, and fragmented chromatins were affinity-purified with streptavidin beads. Enrichment of the *Nanog* promoter in the purified chromatins was determined by qPCR. Enrichment was normalized by fold enrichment of gRNA4-transduced control. Data represent the mean ± SEM of three independent experiments. (**E**) Morphology of EB5/dCNBR cells transduced with gRNA-expressing retroviral vector. After transduction of MLV(U6-gRNA) expressing indicated gRNA, cells were selected by 2 µg/mL puromycin for 2 days. Scale bars, 200 µm. (**F**) *Nanog* expression in cells for CAPTURE of the *Nanog* promoter. *Nanog* mRNA levels in EB5/dCNBR cells with gRNA2 was determined 5 days after puromycin selection. mRNA level was normalized by that of mock-transduced EB5 cells. Data represent the mean ± SEM of three independent experiments. ** *p* < 0.01.

To identify proteins involved in the regulation of gene expression in mouse embryonic stem cells (mESCs), we used CAPTURE, an approach that allows unbiased identification of proteins assembled on a specific chromatin site [19]. The CAPTURE method utilizes binding of biotinylated dCas9 via a guide RNA (gRNA) that targets a specific chromatin site, enabling subsequent affinity purification of the targeted chromatin by streptavidin beads. To use the CAPTURE method, we created a Sendai virus vector, SeVdp(dCNBR), that expresses the biotin ligase BirA and FLAG-tagged dCas9 fused with a biotin acceptor site. SeVdp(dCNBR) also carries genes for neomycin resistance and the fluorescence protein Keima-Red, which allows for the selection and monitoring of infected cells, respectively (Figure 1A). SeVdp(dCNBR) is based upon a replication-defective and persistent Sendai virus vector (SeVdp), which expresses multiple exogenous genes at high levels [20]. SeVdp remains stable in the cytoplasm of infected cells and is free from the silencing that often occurs when a gene is expressed from a retrovirus-based vector in mESCs [21,22].

To test if SeVdp(dCNBR) expresses biotinylated dCas9 stably in mESCs, we infected SeVdp(dCNBR) to mESCs (EB5 cells) and NIH3T3 cells, which were then selected with G418 (Figure 1A). As shown in Figure 1B, the infected mESCs and NIH3T3 showed strong Keima-Red fluorescence, indicating that SeVdp(dCNBR) enables the stable, high-level expression of exogenous genes. Alexa488-conjugated streptavidin, which interacts with biotinylated proteins and emits green fluorescence, showed strong staining in nuclei of the infected cells, indicating that the expressed dCas9 was biotinylated and localized in nuclei (Figure S1A). Indeed, dCas9 was precipitated efficiently from NIH3T3 cells by streptavidin beads (Figure S1B). Thus, SeVdp(dCNBR) stably expresses biotinylated dCas9, which can be subsequently enriched by affinity purification using streptavidin beads.

We then chose the *Nanog* promoter as the target of dCas9 because *Nanog* expression is closely associated with mESC pluripotency [23]. To enable dCas9 to bind the regulatory region of the *Nanog* gene in mESCs, we used the CRISPR direct tool (https://crispr.dbcls.jp/) to design three guide RNAs (gRNA1, 2, and 3) targeting the *Nanog* promoter regions that do not overlap with consensus binding sites for the OCT4/SOX2 complex (Figure 1C). These gRNAs regenerated EGFP through Cas9-mediated digestion and subsequent homologous recombination [24] (Figure S1C), indicating that they recruit the Cas9 protein to their cognate target sequence. After retrovirus-mediated transduction of gRNAs into EB5 cells with SeVdp(dCNBR) (EB5/dCNBR), DNA bound by dCas9 was precipitated by CAPTURE (Figure S1D). ChIP-qPCR assays using the precipitated DNA indicated that the *Nanog* promoter region was enriched by CAPTURE, with gRNA2 showing the highest enrichment (Figure 1D). Specific binding of dCas9 to the *Nanog* promoter regions with three gRNAs did not alter cell morphology of EB5 cells (Figure 1E), and *Nanog* expression did not decrease but rather increased moderately (Figure 1F). Interestingly, NIH3T3 cells showed enrichment of the *Nanog* promoter only with gRNA1 (Figure S1E), suggesting differential dCas9 accessibility to the *Nanog* chromatin between mESCs and fibroblasts.

### 2.2. Isolation of Proteins Assembled on the Endogenous Nanog Chromatin

**Figure 2:**
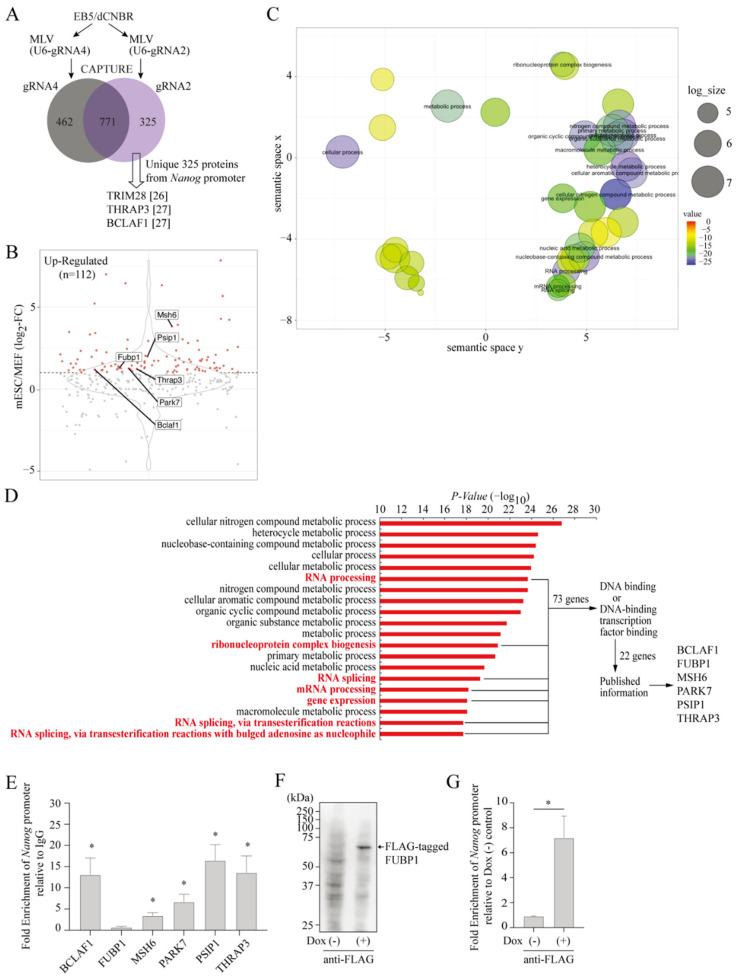
Purification of proteins binding to the *Nanog* promoter in mESCs. (**A**) Venn diagram of identified proteins by LC/MS/MS using gRNA2 and gRNA4. Proteins were identified from three independent experiments. In total, 325 proteins were obtained as unique proteins for gRNA2. (**B**) Differential expression of the unique proteins between mouse embryonic fibroblasts (MEFs) and mESCs. Published RNA-seq data [28] was used to obtain expression data of each unique protein in MEFs and mESCs. A log_2_-fold change was calculated and shown in a violin plot. Highly expressed (>two-fold) genes in mESC were selected for further analyses. The six selected proteins are highlighted. (**C**) Gene ontology (GO) terms of biological processes enriched in the selected genes. GO Term Finder was used for GO analysis of the highly expressed genes in mESCs (n = 112), and the data was visualized in the REViGO using default parameters. (**D**) Top 20 enriched GO terms in GO analysis in (**C**). Seventy-three genes in GO terms related to RNA processing, RNA splicing, ribonucleoprotein complex biogenesis, and gene expression were narrowed down stepwise to select the six proteins. (**E**) Binding of the six selected proteins to the *Nanog* promoter. ChIP assays of chromatin from EB5 cells was performed using an antibody against each protein. Data represent the mean ± SEM of three independent experiments. * *p* < 0.05 versus ChIP with normal mouse IgG. (**F**) Expression of FLAG-tagged FUBP1. EB5 cells were transduced with lentiviral vector (LV(TO-3F-Fubp1)) expressing FLAG-tagged FUBP1 using a doxycycline-inducible expression system. Whole cell lysate from the cells cultured with or without 2 µg/mL doxycycline for 5 days were prepared and subjected to western blotting using anti-FLAG antibody. (**G**) Binding of FUBP1 to the *Nanog* promoter. ChIP assay of chromatin from cells in (**F**) was performed using anti-FLAG antibody. Data are represented as the means ± SEM of three independent experiments. * *p* < 0.05.

To isolate proteins associated with the *Nanog* chromatin in vivo, soluble chromatin samples isolated from EB5/dCNBR cells expressing either gRNA2 or control gRNA (gRNA4) targeting a Gal4 binding sequence [19] were subjected to affinity purification using streptavidin beads (Figure S1D). The proteins retained on the beads were subjected to protease digestion and then analyzed by mass spectrometry. After excluding proteins identified in both gRNA2- and gRNA4-expressing EB5/dCNBR cells, we obtained 325 proteins that occupy the *Nanog* promoter (Figure 2A, Table S1). Out of these 325 proteins, some were previously shown to occupy the *Nanog* promoter, including TRIM28, THRAP3, and BCLAF1 [25,26,27] (Figure 2A), indicating that the CAPTURE procedure successfully enriched proteins from the mESC *Nanog* promoter, which are potentially relevant to mESC functions.

To further confirm the relevance of the identified proteins to mESC functions, we compared mRNA expression levels of the 325 proteins between mouse embryonic fibroblasts (MEFs) and mESCs using published RNA-Seq data [28]. It revealed that nearly 34% (112 proteins) of the proteins have >two-fold higher mRNA expression in mESCs than in MEFs (Figure 2B). When analyzed by Gene Ontology (GO) Term Finder [29] and REViGO [30], the 112 proteins were enriched in functions related to various metabolic processes, RNA processing, RNA splicing, and gene expression (Figure 2C,D). After excluding the metabolic processes, we obtained 73 proteins that were included in GO terms related to RNA processing, RNA splicing, ribonucleoprotein complex biogenesis, and gene expression. Out of the 73 proteins, 22 proteins were selected based upon DNA binding or DNA-binding transcription factor binding (Figure 2D, Table S2). We chose six proteins included in the 22 proteins because of their potential relevance to some aspects of specific gene regulation based upon the published literature: For example, BCLAF1 and THRAP3 are involved in selective pre-mRNA splicing and the export of mRNA [31]; FUBP1 regulates *c-Myc* transcription and pre-mRNA splicing [32,33]; Park7 regulates hypoxia-induced gene expression [34]; PSIP1 plays regulatory roles in transcription and alternative splicing [35,36]. Moreover, a DNA repair protein MSH6 has been shown to cooperate with OCT4 in mouse [37,38] and human mESCs [39].

Because these proteins lack any conventional DNA-binding domain with a sequence-specific binding activity, we performed ChIP-qPCR experiments using an antibody against each protein to confirm if they occupy the *Nanog* chromatin in mESCs. Figure 2E shows that BCLAF1, MSH6, PARK7, PSIP1, and THRAP3 occupy the *Nanog* promoter region in mESCs except for FUBP1, for which the quality of the cognate antibody was poor. For FUBP1, we expressed FLAG-tagged FUBP1 in mESCs using a doxycycline-inducible lentivirus system. As shown in Figure 2F,G, FLAG-tagged FUBP1 was expressed in mESCs and found to occupy the *Nanog* chromatin in a doxycycline-dependent manner.

### 2.3. Effects of CAPTURE-Identified Proteins on mESC Pluripotency

Given that the six proteins lack DNA sequence-specific binding activity and have roles at multiple steps of gene expression, we wondered if they might regulate expression of broader sets of genes associated with pluripotency. Thus, we established mESCs that enables live-cell monitoring of pluripotency independently of changes in *Nanog* expression. To do this, we chose *Rex1*, another gene closely associated with mESC pluripotency, and established mESCs in which Kusabira Orange fluorescence protein (hKO) was integrated into one of the *Rex1* alleles and driven by its promoter (Figures S2A–C). These mESCs, termed EB5/ReKO cells, expressed hKO fluorescence when maintained in mESC medium containing Leukemia Inhibitory Factor (LIF) (Figure S2D, upper panels). However, when retinoic acid was added to mESCs in the absence of LIF, mESC colonies showed flattened morphology, which is indicative of reduced pluripotency, and concomitantly diminished hKO fluorescence (Figure S2D, lower panels). Thus, the EB5/ReKO cells allow live-cell monitoring of *Rex1* expression and enables assessment of changes in pluripotency during self-renewal and differentiation of live mESCs.

**Figure 3:**
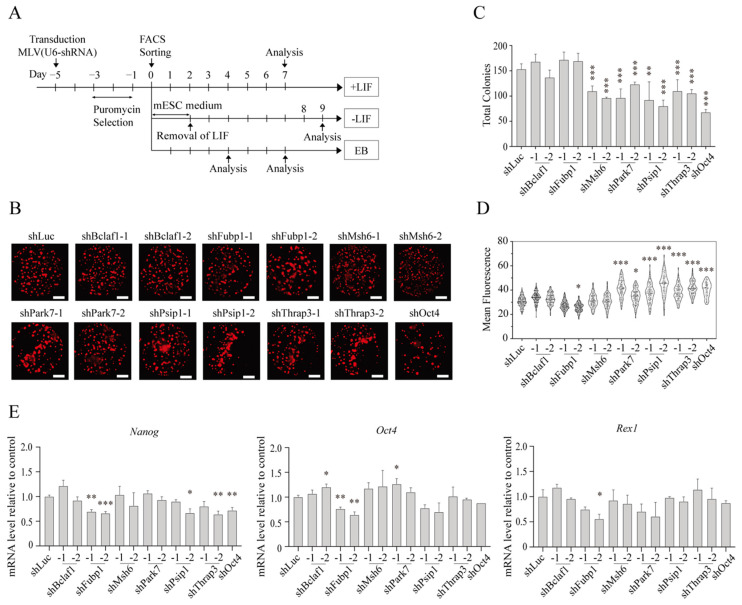
The effect of knockdown of identified proteins on self-renewal and pluripotency of mESCs. (**A**) Experimental scheme of pluripotency and differentiation analyses. EB5/ReKO cells were transduced with retroviral vector expressing each shRNA followed by puromycin selection for 2 days. Five days after transduction, 1000 cells/well were sorted to a flat-bottom 96-well plate for culture in mESC medium with Leukemia Inhibitory Factor (+LIF) or without (-LIF) LIF, or 1 × 10^3^ cells/well to V-bottom 96-well plate for embryoid body (EB) formation (EB). (**B**) Whole well fluorescence images of hKO(+) mESC colonies cultured with LIF. EB5/ReKO cells treated with indicated shRNA and cultured in mESC medium with LIF. Whole well images were collected 7 days after sorting. Representative images are shown. Scale bars, 1500 µm. (**C**) Colony numbers of mESC treated with shRNA. Colony numbers were counted from the images collected in (**B**). Data represent the mean ± SEM from five independent experiments. ** *p* < 0.01, *** *p* < 0.001 versus EB5/ReKO cells treated with control shRNA (shLuc). (**D**) Mean fluorescent intensity of mESCs treated with shRNA. hKO fluorescent intensity in each colony was measured from the images collected in (**B**). Data represent the mean ± SEM from total colonies in each shRNA. * *p* < 0.05, *** *p* < 0.001 versus EB5/ReKO cells treated with control shRNA (shLuc). (**E**) mRNA level of pluripotency-related genes. *Nanog*, *Oct4*, or *Rex1* mRNA levels in the EB5/ReKO cells prepared as (**B**) were determined 7 days after cell sorting. Data represent the mean ± SEM of three independent experiments. * *p* < 0.05, ** *p* < 0.01, *** *p* < 0.001 versus EB5/ReKO cells treated with control shRNA (shLuc).

Next, to test the roles for the identified proteins in mESC pluripotency, we designed two small hairpin RNAs (shRNA) against each identified protein and transduced them into EB5/ReKO cells using a silencing-resistant retrovirus vector (Figure 3A, Figure S3A). The selection of mESCs with puromycin was kept to a minimum (5 days) to prevent significant changes in hKO fluorescence (Figure S3B) as well as in *Nanog* and *Oct4* expression (Figure S3C,D), and 1 × 10^3^ shRNA expressing and hKO(+) EB5/ReKO cells were sorted and allowed to grow in a 96-well plate (Figure 3A, +LIF). Seven days after cell sorting, the number of colonies and the intensity of hKO fluorescence in each colony were determined to assess self-renewal of the cells (Figure S3E). As shown in Figure 3B,C, the number of mESC colonies was reduced upon knockdown of *Msh6, Park7, Psip1*, and *Thrap3*, indicating their possible roles in self-renewal of mESCs. Interestingly, knockdown of *Park7, Psip1*, and *Thrap3* increased the intensity of hKO fluorescence as well (Figure 3B,D), possibly due to the reduced size of individual mESCs. The expression of *Nanog, Oct4*, and *Rex1* showed little or minor changes upon knockdown of *Bclaf1, Msh6, Park7, Psip1*, and *Thrap3*; however, *Fubp1* knockdown showed clear downregulation of *Nanog* and *Oct4*, and possibly of *Rex1*, indicating that FUBP1 is important for maintaining mESC pluripotency (Figure 3D,E). Thus, MSH6, PARK7, PSIP1, and THRAP3 are required for proper self-renewal of mESCs, whereas FUBP1 is required for maintaining the expression of pluripotency genes in mESCs.

### 2.4. Roles for the Identified Proteins in mESC Differentiation

**Figure 4:**
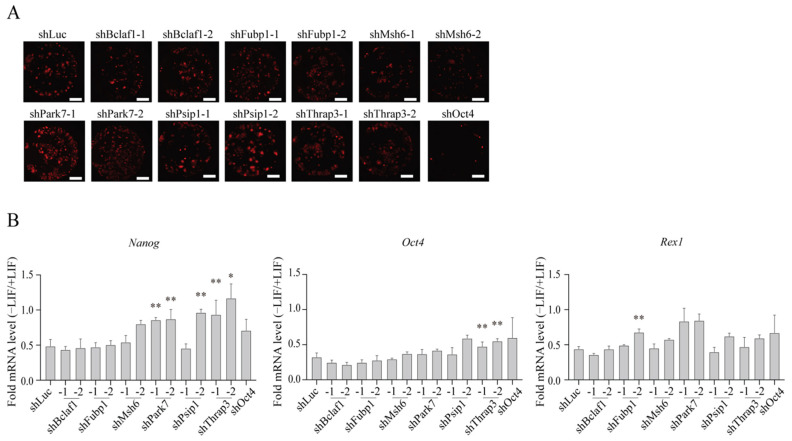
Effect of knockdown of identified proteins on loss of pluripotency. (**A**) Whole well fluorescence images of hKO(+) mESC colonies cultured without LIF. EB5/ReKO cells treated with indicated shRNA and cultured in mESC medium without LIF. Whole well images were collected 9 days after cell sorting. Representative images are shown. Scale bars, 1500 µm. (**B**) mRNA level of pluripotency-related genes. *Nanog*, *Oct4*, or *Rex1* mRNA levels in the EB5/ReKO cells prepared as (**A**) were determined 9 days after cell sorting. Data represent the mean ± SEM of three independent experiments. * *p* < 0.05, ** *p* < 0.01 versus EB5/ReKO cells treated with control shRNA (shLuc).

Despite their demonstrated presence on the *Nanog* promoter (Figure 2E,G), these proteins (except FUBP1) displayed miniscule effects on *Nanog* expression during maintenance of mESCs in culture (Figure 3E). This raised the question of whether the identified proteins are poised for, but not yet actively engaged in regulation of gene expression unless mESCs are allowed to enter the differentiation stage. To explore this possibility, we knocked down each protein and then removed LIF from the medium to allow mESCs to exit from pluripotency. EB5/ReKO cells expressing shRNA for each identified protein, prepared as described previously (Figure S3), were FACS-sorted and plated at 1 × 10^3^ cells per well. After 2 days of culture in the presence of LIF, the cells were maintained for 7 days in mESC medium without LIF (Figure 3A, -LIF). Under this condition, mESCs lost hKO fluorescence gradually (Figure 4A) and downregulated the expression of *Nanog*, *Oct4*, and *Rex1* by 51.6%, 67.8%, and 55.5%, respectively (Figure 4B, shLuc), indicating that mESCs exited from pluripotency. The knockdown of *Park7* diminished the downregulation of *Nanog* expression, and in the case of *Thrap3*, the downregulation of both *Nanog* and *Oct4* expression were diminished, indicating that PARK7 and THRAP3 are required for down regulating *Nanog* and *Oct4* when mESCs exit from pluripotency (Figure 4B).

**Figure 5:**
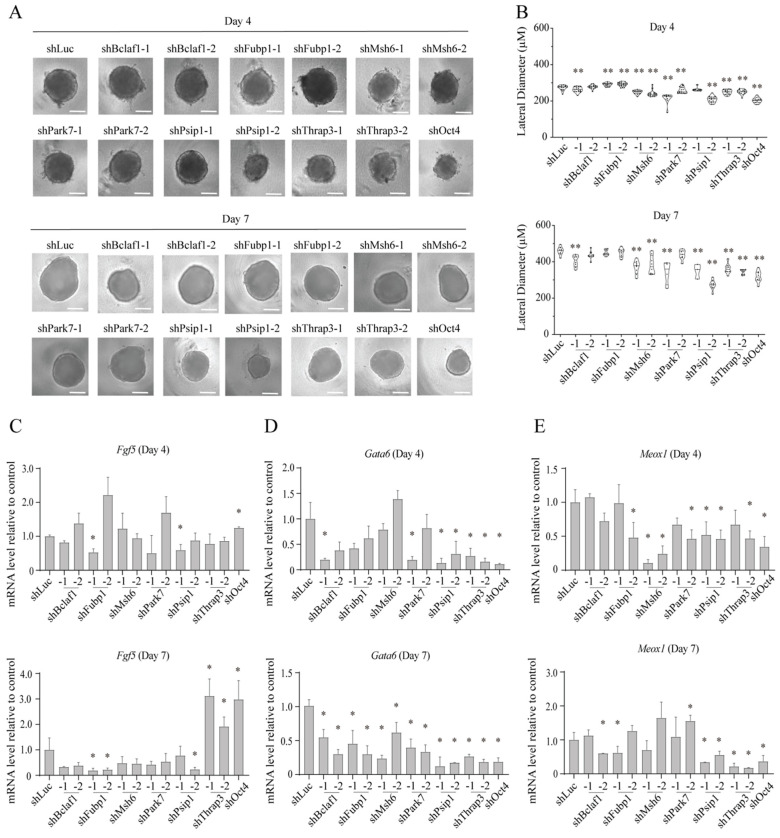
Effect of knockdown of identified proteins on differentiation of mESCs. (**A**) Bright-field images of EBs. Representative image of EB from EB5/ReKO cells treated with indicated shRNA and cultured in EB differentiation medium were taken at the indicated date. Scale bars, 150 µm. (**B**) Size of EBs. Lateral diameters of EBs from EB5/ReKO cells prepared as (**A**) were measured 4 days (upper) or 7 days (lower) after sorting. Data represent the mean ± SEM of 16 (Day 4) or 8 (Day 7) colonies. ** *p* < 0.01 versus EB5/ReKO cells treated with control shRNA (shLuc). (**C**–**E**) mRNA level of lineage marker genes. *Fgf5* (**C**), *Gata6* (**D**), or *Meox1* (**E**) mRNA levels in the EB from EB5/ReKO cells prepared as (**A**) were determined 4 days (upper) or 7 days (lower) after cell sorting. Data represent the mean ± SEM of three independent experiments. * *p* < 0.05 versus EB5/ReKO cells treated with control shRNA (shLuc).

To further explore the roles for these genes during mESC differentiation, we transduced shRNA retroviruses into EB5/ReKO cells and allowed them to differentiate via embryoid body (EB) formation. In this procedure, EBs generate epiblasts and primitive endoderm in vitro, which closely recapitulates formation of the two lineages from inner cell mass in the blastocyte in vivo. Five days after transduction and drug selection, EB5/ReKO cells were FACS-sorted onto non-coated 96-well plates with v-bottom wells to produce EBs of relatively uniform sizes (Figure 5A). Then, the EBs were allowed to grow and differentiate for 4 or 7 days without LIF (Figure 3A, EB). As measured by the lateral diameter, EBs were significantly smaller when *Msh6*, *Psip1*, *Park7* and *Thrap3* were knocked down (Figure 5A,B), indicating their potential roles for differentiation via EB formation. While the expression of an epiblast maker *Fgf5* decreased upon *Fubp1* knockdown, it increased upon *Thrap3* knockdown (Figure 5C). The expression of a primitive endoderm marker *Gata6* was decreased upon knockdown of all six genes, indicating their potential roles in differentiation of primitive endoderm (Figure 5D). Expression of *Meox1*, a gene expressed in mesodermal cells, decreased upon knockdown of *Psip1* and *Thrap3* (Figure 5E). Thus, while all six genes are important for differentiation of primitive endoderm, they may play a role in derivation of limited types of germ layers.

### 2.5. Roles for the Identified Proteins in Somatic Cell Reprogramming

To further corroborate the roles for the identified proteins in pluripotency, we next tested their effects on somatic cell reprogramming, which may be considered as a reversal of mESC differentiation. We previously developed a somatic cell reprogramming system utilizing a Sendai virus vector expressing OCT4, SOX2, KLF4, and c-MYC (SeVdp(fK-OSM)) [40]. The vector expresses KLF4 fused with the destabilization domain (DD), and the reduced level of KLF4 generates partially reprogrammed iPSCs. Because the DD is inhibited by a small chemical Shield1 [41], it stabilizes DD-fused KLF4 and restores the KLF4 level closer to its original level. Distinct levels of KLF4 reprogram MEFs to different extents, and fully reprogrammed iPSCs are generated with 100 nM Shield1 (High-K condition), whereas partially reprogrammed iPSCs are generated without Shield1 (Low-K condition).

**Figure 6:**
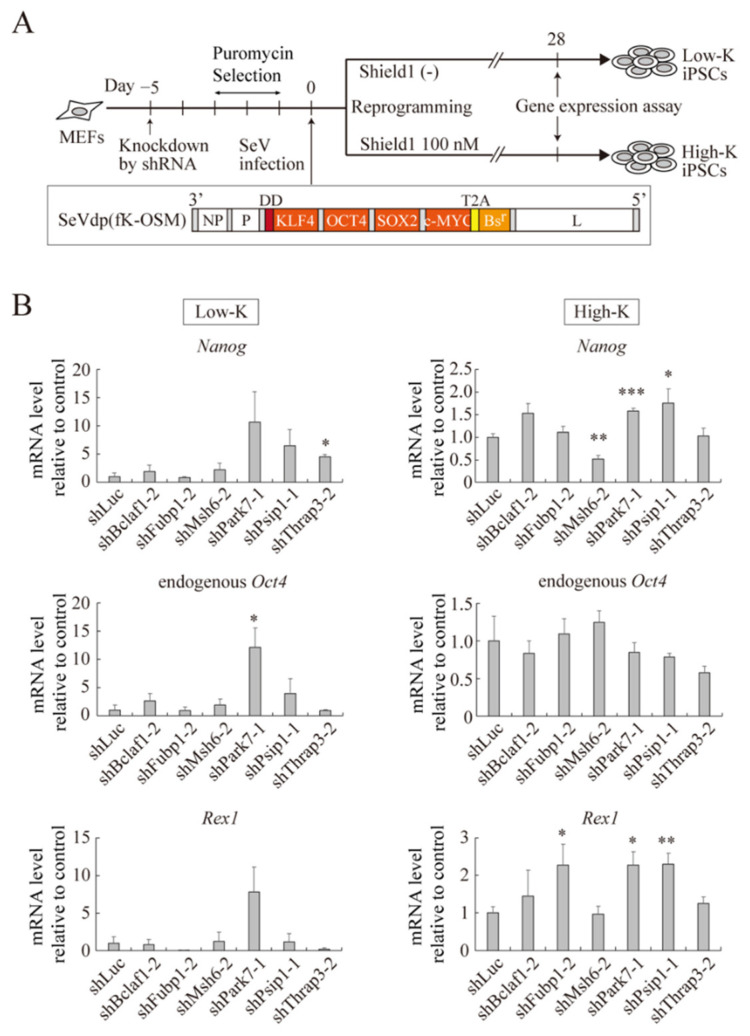
Effect of knockdown of identified proteins on somatic cell reprogramming. (**A**) Schematic representation of iPSC production with knockdown of identified proteins. MEFs were transduced with retroviral vector expressing each shRNA. After puromycin selection for 3 days, the selected MEFs were infected with SeVdp(fK-OSM) and cultured with (High-K) or without (Low-K) 100 nM Shield1. (**B**) mRNA level of pluripotency-related genes. MEFs were treated with indicated shRNA and reprogrammed as described in (**A**). *Nanog*, endogenous *Oct4,* and *Rex1* mRNA levels were determined at day 28 of reprogramming. Data represent the mean ± SEM of three independent experiments. * *p* < 0.05, ** *p* < 0.01, *** *p* < 0.001 versus reprogrammed MEFs treated with control shRNA (shLuc).

We transduced MEFs with retrovirus expressing shRNA against each identified gene and reprogrammed the MEFs under both Low-K and High-K conditions (Figure 6A). Under the Low-K condition, knockdown of *Park7* and *Thrap3* increased the expression of *Oct4* and *Nanog*, respectively (Figure 6B). Although statistically not significant, knockdown of *Park7* appeared to increase the expression of *Nanog* and *Rex1* as well (Figure 6B). Under the High-K condition, knockdown of *Park7* and *Psip1* significantly upregulated *Nanog* expression, whereas *Msh6* knockdown reduced *Nanog* expression (Figure 6B). In addition, knockdown of *Fubp1, Park7*, and *Psip1* significantly increased *Rex1* expression (Figure 6B). Thus, consistent with the enhancing effect of *Park7, Psip1*, and *Thrap3* on mESC self-renewal or differentiation, they act as roadblocks of somatic cell reprogramming.

## 3. Discussion

Here, we have purified the proteins associated with the *Nanog* promoter region in mESCs and identified FUBP1 as an essential factor for the expression of pluripotency genes in mESCs and PARK7, PSIP1, and THRAP3 as positive regulators of mESC self-renewal or differentiation as well as roadblock factors of somatic cell reprogramming. The CAPTURE method enabled enrichment of the *Nanog* chromatin for subsequent mass spectrometric identification of resident chromatin proteins, which were confirmed by ChIP-qPCR to occupy the *Nanog* promoter region. Given the experimentally confirmed occupancy of these factors on the *Nanog* promoter region as well as inclusion of the previously reported factors such as TRIM28, THRAP3, and BCLAF1 among the identified proteins, the CAPTURE method appears to provide sufficient enrichment for the locus-specific isolation of the mESC chromatin.

In our CAPTURE experiments, we failed to identify well-characterized transcription factors including OCT4 and SOX2, which are known to directly bind the regulatory regions of the *Nanog* gene. Given the high enrichment required for comprehensively isolating a single-locus chromatin from cells [42], a more scalable mESC culture system should be employed. In addition, further improvement in fold enrichment may be necessary to identify DNA-binding transcription factors associated with a single genomic locus. Indeed, a recent study reported a CAPTURE 2.0 system that has even higher enrichment [43]. This redesigned CAPTURE 2.0 system may be better suited for isolating a single-locus chromatin in mESCs than the original CAPTURE system that we used here. Based on the analysis of *Nanog* expression, we inferred that dCas9 bound the region that does not have a negative effect on *Nanog* expression (Figure 1E,F). However, given that *Nanog* expression is increased moderately (Figure 1F), dCas9 could have changed the binding pattern of transcription factors. Thus, it may be necessary to carefully select dCas9 target sites that are readily accessible but devoid of functionally relevant transcription factors that affect *Nanog* expression.

Despite these caveats, we could identify functionally relevant proteins from the *Nanog* chromatin in mESCs using the CAPTURE method (Figure 2). Most of the identified proteins (BCLAF1, FUBP1, PSIP1, PARK7, and THRAP3) have been shown to interact with RNA or RNA-associated proteins and are involved in multiple RNA-related cellular processes [36,44]. For instance, PSIP1, THRAP3, and BCLAF1 interact with components of alternative RNA splicing [31,36]. This predominant identification of RNA-binding proteins (RBPs) may be due to their abundance as compared with canonical sequence specific DNA-binding factors. Moreover, RBPs may have remained tethered to the *Nanog* chromatin via RNA transcripts even when dCas9 formed an R-loop in the target DNA [45] and possibly evicted DNA-binding factors from the adjacent *Nanog* chromatin. Although RBPs may be abundant and tethered indirectly to the chromatin, the pathway analysis of the identified factors strongly supports the functional relevance of these RBPs to the gene expression of mESCs (Figure 2). Indeed, our functional assay shows that they have important and probably specific functions during mESC differentiation and somatic cell reprogramming.

Besides transcriptional and epigenetic regulations involving the core pluripotency network, recent studies emphasize the significant roles for RBPs in mESC functions [46,47,48,49,50]. The functions of RBPs in pluripotency and reprogramming include not only splicing, polyadenylation, mRNA stability, and translation but also epigenetic regulation and RNA modification [51]. In particular, the notable roles of alternative splicing in mESC functions [47,52,53] are consistent with our identification of PSIP1, THRAP3, and BCLAF1, which interact with components of alternative RNA splicing [31,36]. Although specific binding of each RBP to RNA is not well defined, recent studies show that RBPs are remarkably specific in executing their functions even when RBPs alone do not show specific RNA-binding [54]. It may be possible that RNA-binding complexes require multiple RBPs to acquire binding specificity toward RNAs. Thus, identifying the whole complexes in an unbiased method such as described here should provide valuable insights for understanding the specific functions of RBPs in mESCs.

## 4. Materials and Methods

### 4.1. Cell Culture

mESCs were cultured in mESC medium (DMEM high Glucose (Nacalai Tesque, Kyoto, Japan) supplemented with 15% Fetal Bovine Serum (FBS) (Thermo Fisher Scientific, Waltham, MA), 100 mM Non-Essential Amino Acids (Nacalai Tesque, Kyoto, Japan), 0.5 mM StemSure Monothioglycerol Solution (FUJIFILM Wako Pure Chemical, Osaka, Japan), 1000 U/mL LIF (FUJIFILM Wako Pure Chemical, Osaka, Japan), and 100 U/mL Penicillin/Streptomycin solution (Nacalai Tesque, Kyoto, Japan)). To form EBs, mESCs were cultured in an EB differentiation medium (DMEM high Glucose supplemented with 20% FBS, 100 mM Non-Essential Amino Acids, 0.5 mM StemSure Monothioglycerol Solution, and 100 U/mL Penicillin/Streptomycin solution). NIH3T3 cells, HEK293T cells, and MEFs were cultured in DMEM medium (DMEM high Glucose supplemented with 10% FBS and 100 U/mL Penicillin/Streptomycin solution). mESCs were allowed to differentiate by culturing the cells in the DMEM medium with 5 µM of retinoic acid (Sigma-Aldrich, St. Louis, MO, USA).

### 4.2. Plasmid Construction

To prepare cDNA for SeVdp(dCNBR), cDNAs of FLAG-tagged dCas9 with a biotin acceptor site, the neomycin-resistant gene (NeoR), *E. coli* biotin ligase (BirA), and Keima-Red were amplified from pEF1a-FB-dCas9-puro (Addgene, Watertown, MA, USA), pcDNA3 (Thermo Fisher Scientific, Waltham, MA, USA), pEF1a-BirA-V5-neo (Addgene, Watertown, MA, USA), and cDNA of SeVdp(KR/Bsr/EGFP/KO) [20], respectively. The amplified cDNAs were inserted into SeVdp vector cDNA using an In-Fusion HD Cloning Kit (TaKaRa Bio, Shiga, Japan). To prepare plasmids or retroviral vectors expressing gRNA for CAPTURE method, gRNA sequences designed by using CRISPR direct (https://crispr.dbcls.jp/) were inserted to pX330-U6-Chimeric_BB-CBh-hSpCas9 (Addgene, Watertown, MA, USA) or pMCs∆YY1-U6-Puro plasmid [55], respectively. pMCs∆YY1-U6-Puro was also used to create a retroviral vector expressing shRNA (MLV(U6-shRNA)) as described previously [55]. Sequences of shRNA were designed using BLOCK-iT™ RNAi Designer (Thermo Fisher Scientific, Waltham, MA, USA). For a lentiviral vector expressing *Fubp1*, the mouse *Fubp1* gene fused with a FLAG-tag was cloned by PCR into pCW57.1 (Addgene, Watertown, MA, USA).

To produce *Rex1* reporter mESCs by genome editing, a donor plasmid was prepared from pJ151-HDR (Addgene, Watertown, MA, USA) which contains a loxP-flanked cassette encoding the puromycin resistance gene (PuroR), the Venus fluorescence marker, and the thymidine kinase (TK) suicide gene. We inserted 5’ UTR of the mouse *Rex1* mRNA (1027 bp) followed by humanized Kusabira Orange (hKO) gene linked with an internal ribosome entry site (IRES) and the zeocin resistant gene (ZeoR) before the loxP-flanked cassette of pJ151-HDR, and 3’ UTR (1717 bp) of *Rex1* mRNA was inserted after the cassette. A sequence of each gRNA targeting just after the start codon of *Rex1* open reading frame was inserted to pX330-U6-Chimeric_BB-CBh-hSpCas9. DNA oligonucleotides for the plasmid constructions are listed in Table S3.

### 4.3. Production and Infection of Viral Vectors

The SeVdp-based vector was prepared as described previously [20]. The SeVdp vector was infected to cells by an incubation at 32 °C for 14–16 h. Drug selection by G418 (Nacalai Tesque, Kyoto, Japan) (800 µg/mL for mES cells and 1000 µg/mL for NIH3T3 cells) was started from day 2 of the infection. The retroviral vector (MLV(U6-gRNA) or MLV(U6-shRNA)) and lentiviral vector (LV(TO-3F-Fubp1)) were prepared as described previously [21]. For the transduction of the retroviral vector or lentiviral vector, cells were cultured in 1:1 mixture of the vector stock and medium containing 8 µg/mL Polybrene (Nacalai Tesque, Kyoto, Japan) for 14–16 h. Drug selection by puromycin (FUJIFILM Wako Pure Chemical, Osaka, Japan) (2 µg/mL) was started from day 2 of the transduction. In case of mESCs, the cells and vector were centrifuged at 1500 rpm for 40 min at RT before the start of the cell culture.

### 4.4. CAPTURE of the Nanog Promoter

#### 4.4.1. Isolation of Chromatin Containing the *Nanog* Promoter

To investigate the efficiency of each gRNA, pX330-U6-Chimeric_BB-CBh-hSpCas9 expressing each gRNA and EGFP donor plasmid pCAG-EGxxFP (Addgene, Watertown, MA, USA) with 409 bp of *Nanog* promoter region amplified by primers listed in Table S3 were transfected to HEK293T cells by Lipofectamine 2000 (Thermo Fisher Scientific, Waltham, MA, USA), and EGFP expression was analyzed 3 days after the transfection.

About 1.0 × 10^8^ of EB5/dCNBR or 3T3/dCNBR cells transduced with MLV(U6-gRNA2) or MLV(U6-gRNA4) were cross-linked with 1% formaldehyde for 10 min at RT, followed by quenching with 0.125 M glycine for 5 min at RT, and then washed twice with ice-cold PBS. To isolate chromatin, the cross-linked cells were resuspended in 10 mL of Cell lysis buffer (25 mM Tris-HCl (pH 7.4), 85 mM KCl, 0.1% Triton X-100, 1 mM DTT, and proteinase inhibitor cocktail (Sigma-Aldrich, St. Louis, MO, USA)) and rotated for 15 min at 4°C. Nuclei were isolated by centrifugation at 2300× *g* for 5 min at 4 °C. The pellet was suspended in 5 mL Nuclear lysis buffer (50 mM Tris-HCl (pH 7.4), 10 mM EDTA, 4% SDS, 1 mM DTT, and proteinase inhibitor cocktail) and incubated for 10 min at RT. The nuclear suspension was then mixed with 15 mL of 8 M urea and centrifuged at 16,100× *g* for 25 min at RT, followed by washing twice in the Nuclear lysis buffer mixed with urea. Pelleted chromatin was then washed twice with 5 mL Cell lysis buffer. The chromatin pellet was resuspended in 5 mL of IP binding buffer (20 mM Tris-HCl (pH 7.4), 1 mM EDTA, 0.1% NP-40, 10% glycerol, and proteinase inhibitor cocktail) without NaCl and then sonicated by a Sonifier 450 (Emerson, St. Louis, MO, USA) to DNA fragments with an average size ∼500 bp (10% amplitude, 0.5 s on 1 s off for 1 min). Fragmented chromatin was collected from the supernatant after centrifugation at 16,100× *g* for 25 min at 4 °C, and NaCl was added to the supernatant to 150 mM. After washing streptavidin magnetic beads (Thermo Fisher Scientific, Waltham, MA, USA) with IP binding buffer three times, the soluble chromatin was added to the beads and incubated overnight at 4 °C. After washing 5 times with IP binding buffer, the beads were subjected to CAPTURE-qPCR or -Proteomics.

#### 4.4.2. CAPTURE-ChIP-qPCR

After overnight incubation and washing, the streptavidin beads were eluted using 80 μL of SDS elution buffer (1% SDS, 10 mM EDTA, 50 mM Tris-HCl, pH 8.0) and incubated at 85 °C for 10 min. Eluted chromatin was separated from beads using a magnetic stand and incubated at 65 °C overnight to reverse cross-linking. DNA fragments were purified by Phenol/Chloroform and ethanol precipitation from the chromatin and subjected to qPCR using primers listed in Table S4.

#### 4.4.3. CAPTURE-Proteomics

For western blotting, the beads were treated with Gel loading buffer (50 mM Tris-HCl (pH 6.8), 2% SDS, 10% glycerol, 0.1 mg/mL bromophenol blue, 5% 2-mercaptoethanol) at 100°C for 5 min, and the eluted proteins were subjected to SDS-PAGE and western blotting as described previously [40] using anti-FLAG antibody (Sigma-Aldrich, St. Louis, MO, USA).

For proteomics analysis, the beads were washed twice in IP binding buffer without NP-40 and treated with trypsin (Promega, Madison, WI, USA) overnight at 37 °C, followed by purification using SPE C-TIP-T300 (FUJIFILM Wako Pure Chemical, Osaka, Japan). A peptide analysis by was performed by LC/MS/MS as described previously [21].

#### 4.4.4. Gene Ontology Analysis

mRNA expression profiles of proteins identified by the proteomics were obtained from published data [28], and the mESC/MEF ratio of the profiles was plotted by a custom R script that is available on GitHub (https://github.com/akikuno/iPS-proteomics (accessed on 12 May 2022)). Proteins expressing highly in mESCs (mESC/MEF > 2) were selected for further analyses. GO Term Finder [29] was used for GO analysis of the selected proteins, and the data was visualized in REViGO [30] using default parameters.

### 4.5. Genome Editing

The donor plasmid and the plasmid expressing Cas9 and the gRNA targeting to *Rex1* locus, listed in Table S3, were transfected to EB5 cells followed by puromycin selection (2 µg/mL). The selected clones were subjected to genotyping to isolate knock-in mESCs, EB5/ReKO-Puro. Next, EB5/ReKO-Puro was transfected with pCAGGS-nisCre plasmid (kindly gifted from Seiya Mizuno at the University of Tsukuba) to remove the loxP-flanked cassette. After ganciclovir (FUJIFILM Wako Pure Chemical, Osaka, Japan) selection (1 µg/mL), *Rex1* reporter mESCs, EB5/ReKO, were isolated from the ganciclovir-resistant clones by genotyping.

Genome DNA was isolated by treatment with Genome DNA isolation buffer (10 mM Tris-HCl (pH8.0), 150 mM NaCl, 10 mM EDTA-NaOH, and 0.1% SDS, 50 µg/mL RNaseA) for 1 h at 37 °C followed by 0.2 mg/mL Proteinase K for 4 h at 65 °C. Genome DNA was purified by phenol/chloroform treatment and ethanol precipitation, and then used for genotyping PCR with primers listed in Table S4.

### 4.6. Cell Sorting and Image Analysis

mESCs were trypsinized to make a single cell suspension and supplemented with 2 µg/mL Propidium Iodide (PI) (Nacalai Tesque, Kyoto, Japan) to distinguish between live and dead cells. Live hKO(+) cells were sorted to a flat- or V-bottom 96-well plate for mESC culture or EB formation, respectively, by MoFlo XDP (Beckman Coulter, Brea, CA, USA).

Whole well images of the 96-well plate were obtained by multi-point capture of BZ-X710 (Keyence, Osaka, Japan). mESC colonies were extracted from the image using the Image Analysis program (Keyence, Osaka, Japan) under the following conditions. First, the measurement area was set by using a circular selection covering the whole well. Second, to remove background, thresholds of hKO fluorescence were set as follows: hue: 255, brightness: 40–125, tolerance: 20. Third, after smoothing the edges, extracted regions whose area was larger than 1500 µm^2^ were defined as colonies. Then, colony numbers and the diameter and brightness of each colony were measured. The lateral diameter of EBs were also measured using the Image Analysis program in BZ-X710.

### 4.7. Gene Expression Analysis

To determine mRNA expression, RNA was extracted by an RNeasy Mini Kit (QIAGEN, Hilden, German) according to the manufacturer’s instructions. Reverse transcription and quantitative PCR (qPCR) were performed as described previously [56]. The expression levels were normalized against that of TATA-box binding protein (TBP). The DNA sequences for the primers are listed in Table S4.

To detect biotin-labeled protein under a microscope, cells were fixed with 3.7% formaldehyde in phosphate buffered saline (PBS). After permeabilization in 0.1% Triton X-100/PBS, cells were stained with Alexa488-conjugated streptavidin (Thermo Fisher Scientific, Waltham, MA, USA). Nuclei were counterstained with 4′,6-diamidino-2-phenylindole (DAPI) using Fluoro-KEEPER Antifade Reagent (Nacalai Tesque, Kyoto, Japan).

### 4.8. Somatic Cell Reprogramming

MEFs were first transduced with a retroviral vector expressing shRNA and then reprogrammed by infection with SeVdp(fK-OSM) as described previously [57].

### 4.9. Statistical Analysis

Student’s t-tests were employed to determine a statistically significant difference between data sets. In the case of whole well image analyses, significance was tested using one way ANOVA in GraphPad Prism v8.0. A value of *p* < 0.05 was regarded as statistically significant.

## Data Availability

The data reported here are available upon reasonable requests. The accession number for the proteomics data reported in this paper is JPST001843 on JPOST (https://jpostdb.org).

## Supplementary Materials

The following supporting information can be downloaded at: https://www.mdpi.com/article/10.3390/ijms232315242/s1.
