# Supplementary material for: Locus-Specific Isolation of the Nanog Chromatin Identifies Regulators Relevant to Pluripotency of Mouse Embryonic Stem Cells and Reprogramming of Somatic Cells
Source: Int J Mol Sci. 2022 Dec 3;23(23):15242. doi: 10.3390/ijms232315242 (PMC9740452; doi:10.3390/ijms232315242)
Supplement: Supplementary file 1 [file ijms-23-15242-s001.zip › ijms-2063209-supplementary.pdf]

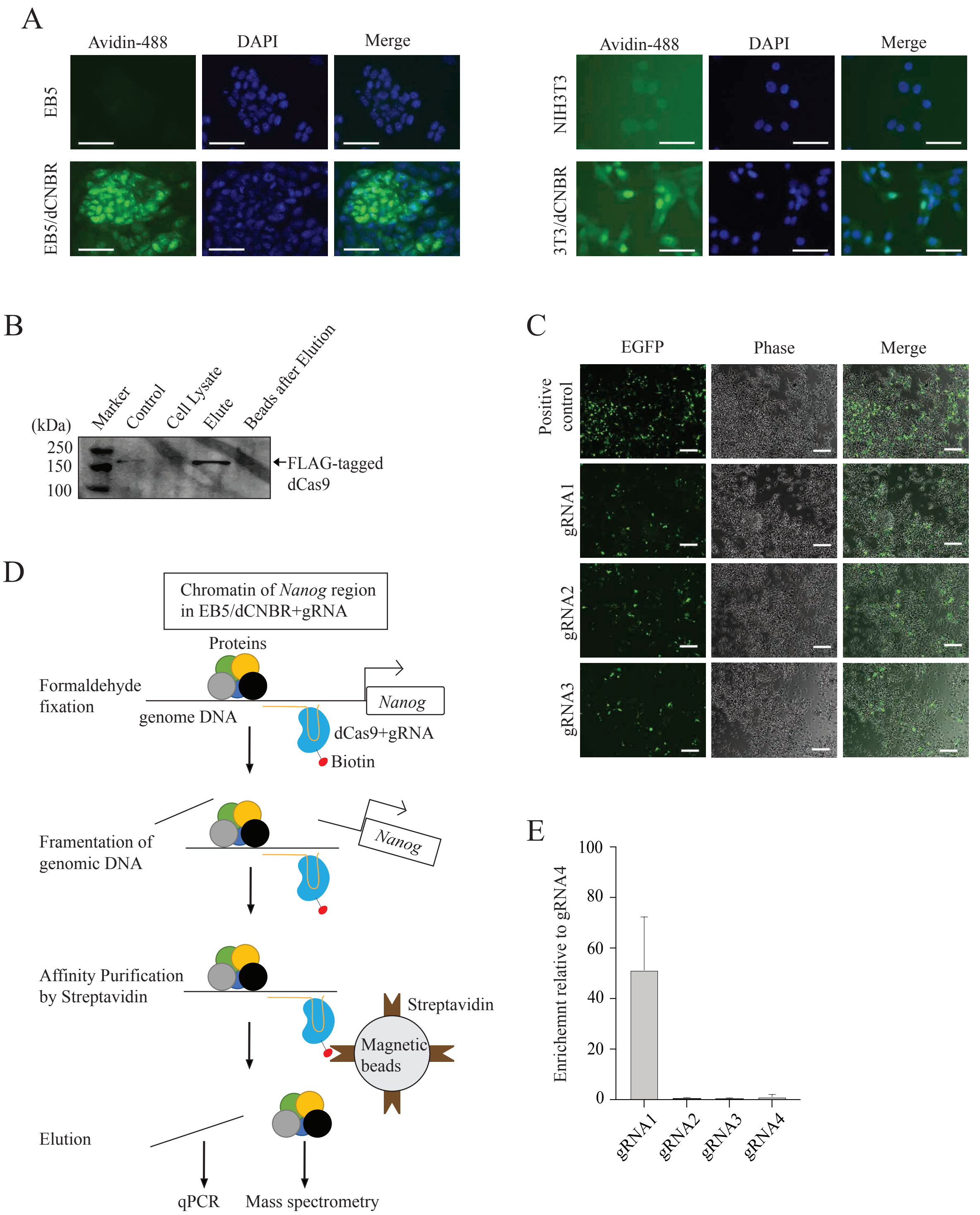

Figure S1. Preparation of the CAPTURE components. (**A**) Expression of biotin-labeled dCas9 from SeVdp(dCNBR). Mock or SeVdp(dCNBR)-infected EB5 or NIH3T3 cells were labeled with Alexa Fluor 488-conjugated Avidin. Scale bars, 50 µm. (**B**) Enrichment of biotin-labelled dCas9 protein using streptavidin beads. Whole cell lysate was prepared from 3T3/dCNBR cells and treated with streptavidin beads. Cell lysate, eluted product, and remained protein on beads after elution were subjected to western blot using anti-FLAG antibody. Cell lysate from NIH3T3 cells transfected with a plasmid expressing FLAG-tagged Cas9 was used as control. (**C**) Efficiency of gRNAs. HEK293T cells were transfected with a plasmid expressing indicated gRNA and Cas9 as well as a donor plasmid spanning *Nanog* promoter region in EGFP ORF. EGFP expression was observed 3 days after transfection. We also used previously described gRNA and its target sequence [31] as positive control. Scale bars, 50 µm. (**D**) Schematic representation of the CAPTURE method of the *Nanog* promoter. Chromatin from EB5/dCNBR cells with gRNA2 or control gRNA (gRNA4) were isolated and fragmented. Then, DNA-protein complexes were affinity-purified by streptavidin beads and analyzed either in qPCR or mass spectrometry. (**E**) Enrichment of *Nanog* promoter DNA by CAPTURE with gRNAs. Chromatins were collected from 3T3/dCNBR cells expressing the indicated gRNA, and fragmented chromatins were affinity purified with streptavidin beads. Enrichment of the *Nanog* promoter in the purified chromatins was calculated by qPCR for *Nanog* promoter. Enrichment was normalized by fold enrichment of gRNA4-transduced control. Data are represented as the means ± SEM of three independent experiments.

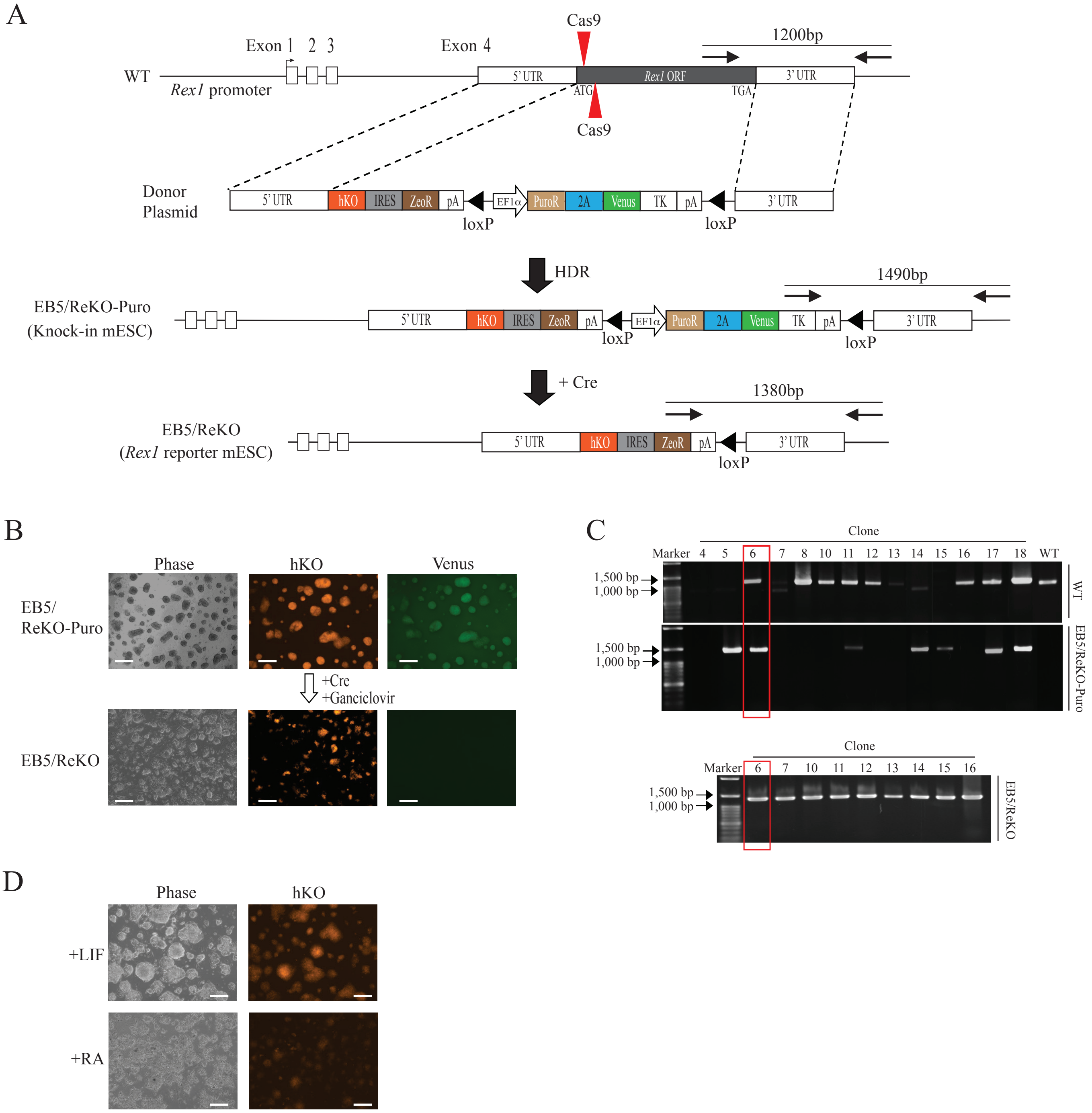

Figure S2. Generation of pluripotency monitoring mESCs. (**A**) Schematics of a production of *Rex1* monitoring mESC. Plasmid encoding Cas9 and gRNA targeting to exon 4 of *Rex1* locus was transfected to EB5 cells. Donor plasmid containing homologous region including 5'- and 3'-UTR and encoding Kusabira Orange (hKO), Zeocin resistant gene (ZeoR), Puromycin resistant gene (PuroR), Venus, and Thymidine kinase (TK) is also transfected at the same time. Knock-in cells undergoing homologous DNA repair (HDR) were selected by Puromycin. After cloning of puromycin-resistant cells, their genomic DNAs were subjected to genotyping to find EB5/ReKO-Puro cells. Then, Cre recombinase was expressed in EB5/ReKO-Puro cells to remove PuroR, Venus, and TK which locate between loxP sequence. Genome DNAs from ganciclovir-resistant clones were used for final genotyping to confirm production of *Rex1* reporter mESC (EB5/ReKO). (**B**) Fluorescent gene expression in knock-in EB5 cells. hKO and Venus expression were investigated in EB5/ReKO-Puro and EB5/ReKO after puromycin selection for 10 days and ganciclovir treatment for 5 days, respectively. Scale bars, 200 µm. (**C**) Genotyping knock-in EB5 cells. Genome DNA from puromycin-resistant clones were collected as indicated in (**A**) and subjected to genotyping PCR with primers for wild type (WT) and EB5/ReKO-Puro. EB5/ReKO-Puro clone 6 was used for further step. Genome DNA from ganciclovir-resistant clones were collected as indicated in (**A**) and subjected to PCR with primers for EB5/ReKO. Finally, EB5/ReKO clone 6 was selected as *Rex1* reporter mESC. (**D**) Expression change of reporter gene according to pluripotency. EB5/ReKO clone 6 was cultured in mESC medium (+LIF) or DMEM medium plus 5 µM of retinoic acid (+RA) for 7 days. Scale bars, 100 µm.

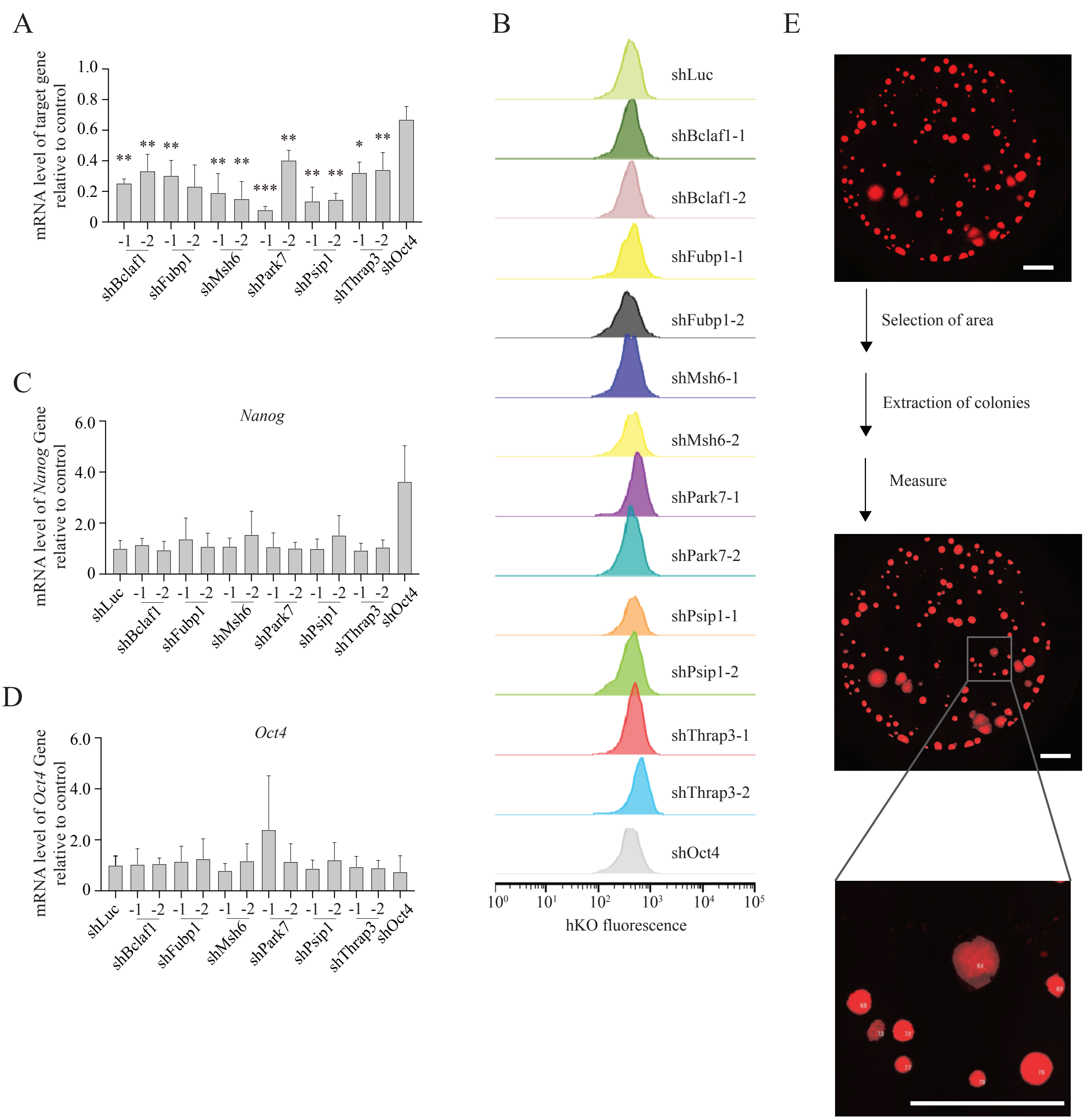

Figure S3. Analyses of mESC with knockdown of identified proteins. (**A**) Suppression of each target gene expression by shRNA. EB5/ReKO cells were transduced with retroviral vector expressing the indicated shRNA followed by puromycin selection. mRNA levels of each target gene were determined 5 days after transduction. Data represent mean ± SEM from three independent experiments. *P < 0.05, **P < 0.01, ***P < 0.001 versus EB5 cells treated with control shRNA (shLuc). (**B**) hKO fluorescent level in *Rex1* reporter cells with shRNA treatment. EB5/ReKO cells were transduced as (**A**). Five days after transduction, cells were analyzed for hKO expression using MoFlo XDP. Representative FACS histgrams are shown. (**C, D**) Pluripotency of cells after shRNA treatment for 5 days. EB5/ReKO cells were transduced as (**A**). *Nanog* (**C**) or *Oct4* (**D**) mRNA levels in the cells were determined 5 days after transduction. Data are represented as the means ± SEM of three independent experiments. (**E**) Pipeline for image analyses of mESC colonies. mESC colonies expressing hKO were analyzed as described in Materials and Methods. Scale bars, 1,000 μm.

Table S1. List of proteins identified by CAPTURE

Accession	Gene Symb	ESC/MEF
Q8BGQ7	Aars	< 2.0
Q5SWU9	Acaca	< 2.0
E9Q4Z2	Acacb	> 2.0
Q07417	Acads	< 2.0
Q9JIX8	Acin1	> 2.0
Q99KI0	Aco2	< 2.0
Q32MW3	Acot10	< 2.0
Q9R0X4	Acot9	< 2.0
P68134	Acta1	< 2.0
P62737	Acta2	< 2.0
P60710	Actb	< 2.0
P68033	Actc1	> 2.0
P63260	Actg1	< 2.0
P45376	Akr1b3	< 2.0
P07724	Alb	< 2.0
O55143	Atp2a2	< 2.0
P56480	Atp5b	> 2.0
Q9DB20	Atp5o	< 2.0
P28658	Atxn10	< 2.0
Q8CI61	Bag4	> 2.0
Q8K019	Bclaf1	> 2.0
P80317	Cct6a	NA
Q61390	Cct6b	> 2.0
P42932	Cct8	> 2.0
Q61081	Cdc37	< 2.0
Q6A068	Cdc5l	> 2.0
P18760	Cfl1	< 2.0
Q68FD5	Cltc	< 2.0
P08122	Col4a2	< 2.0
Q9QZS0	Col4a3	< 2.0
O55029	Copb2	< 2.0
Q9ERK4	Cse1l	> 2.0
P16381	D1Pas1	< 2.0
Q9JII5	Dazap1	> 2.0
Q501J6	Ddx17	< 2.0
Q9JIK5	Ddx21	> 2.0
Q8VDW0	Ddx39	> 2.0
Q9Z1N5	Ddx39b	> 2.0
Q62167	Ddx3x	> 2.0
Q61656	Ddx5	< 2.0
O35286	Dhx15	< 2.0
Q9D2G2	Dlst	< 2.0
Q9QYJ0	Dnaja2	< 2.0
Q9CWR8	Dnmt3l	< 2.0
P10126	Eef1a1	> 2.0
P62631	Eef1a2	< 2.0
O70251	Eef1b2	< 2.0
Q9D8N0	Eef1g	< 2.0
P58252	Eef2	< 2.0
O08810	Eftud2	> 2.0
P63242	Eif5a	> 2.0
Q8BGY2	Eif5a2	< 2.0
P17182	Eno1	> 2.0
P17183	Eno2	< 2.0
P21550	Eno3	< 2.0
Q9DCW4	Etfb	< 2.0
P62862	Fau	< 2.0
P35550	Fbl	> 2.0
Q80WS3	Fbll1	< 2.0
P39749	Fen1	> 2.0
P97807	Fh1	< 2.0
P30416	Fkbp4	> 2.0
P11276	Fn1	< 2.0
Q91WJ8	Fubp1	> 2.0
P97855	G3bp1	> 2.0
P16858	Gapdh	> 2.0
P03975	Gfap	< 2.0
Q61543	Glg1	< 2.0
P50247	Gm4737	NA
P05202	Got2	< 2.0
P24472	Gsta4	> 2.0
P10922	H1f0	< 2.0
Q8R1M2	H2afj	< 2.0

Accession	Gene Symb	ESC/MEF
Q3THW5	H2afv	> 2.0
P27661	H2afx	> 2.0
P0C0S6	H2afz	> 2.0
P84244	H3f3a	< 2.0
P02301	H3f3c	NA
P01942	Hba-a1	< 2.0
Q6NVF4	Helb	> 2.0
P43275	Hist1h1a	> 2.0
P43276	Hist1h1b	< 2.0
P15864	Hist1h1c	< 2.0
P43277	Hist1h1d	< 2.0
P43274	Hist1h1e	< 2.0
Q07133	Hist1h1t	< 2.0
C0HKE4	Hist1h2ac	< 2.0
Q8CGP5	Hist1h2af	> 2.0
Q8CGP6	Hist1h2ah	> 2.0
Q8CGP7	Hist1h2ak	> 2.0
P70696	Hist1h2ba	< 2.0
P68433	Hist1h3g	> 2.0
Q6GSS7	Hist2h2aa1	< 2.0
Q64522	Hist2h2ab	< 2.0
Q64523	Hist2h2ac	< 2.0
Q64524	Hist2h2be	< 2.0
Q8BFU2	Hist3h2a	< 2.0
Q9D2U9	Hist3h2ba	> 2.0
Q8CGP0	Hist3h2bb-p	< 2.0
P17095	Hmga1	< 2.0
P52927	Hmga2	< 2.0
P63158	Hmgb1	< 2.0
P30681	Hmgb2	> 2.0
O54879	Hmgb3	< 2.0
P09602	Hmgn2	< 2.0
O88569	Hnrnpa2b1	< 2.0
Q99020	Hnrnpab	> 2.0
Q9Z204	Hnrnpc	> 2.0
P61979	Hnrnpk	< 2.0
Q8R081	Hnrnpl	> 2.0
Q9D0E1	Hnrnpm	> 2.0
Q8VEK3	Hnrnpu	> 2.0
P07901	Hsp90aa1	> 2.0
P11499	Hsp90ab1	> 2.0
P08113	Hsp90b1	< 2.0
Q61696	Hspa1a	< 2.0
P17879	Hspa1b	< 2.0
P16627	Hspa1l	< 2.0
P17156	Hspa2	< 2.0
P63017	Hspa8	> 2.0
P14602	Hspb1	> 2.0
P63038	Hspd1	> 2.0
P54071	Idh2	< 2.0
P33175	Kif5a	> 2.0
Q61768	Kif5b	< 2.0
P28738	Kif5c	< 2.0
P04104	Krt1	< 2.0
P08730	Krt13	> 2.0
Q61781	Krt14	> 2.0
Q61414	Krt15	< 2.0
Q9QWL7	Krt17	< 2.0
P19001	Krt19	< 2.0
Q3TTY5	Krt2	< 2.0
Q8K0Y2	Krt33a	< 2.0
Q9D646	Krt34	< 2.0
Q497I4	Krt35	< 2.0
Q6IFX3	Krt40	< 2.0
Q6IFX2	Krt42	< 2.0
Q922U2	Krt5	< 2.0
Q9R0H5	Krt71	< 2.0
Q6NXH9	Krt73	< 2.0
Q6IFZ9	Krt74	< 2.0
Q3UV17	Krt76	< 2.0
Q6IFZ6	Krt77	< 2.0
Q8VED5	Krt79	< 2.0
P11679	Krt8	> 2.0

Accession	Gene Symb	ESC/MEF
Q9ERE2	Krt81	< 2.0
Q99M74	Krt82	< 2.0
P97861	Krt86	< 2.0
P19137	Lama1	> 2.0
P02469	Lamb1	< 2.0
P48678	Lmna	< 2.0
P14733	Lmnb1	> 2.0
Q9D5S7	Lrguk	< 2.0
Q8BGS0	Mak16	> 2.0
P63085	Mapk1	< 2.0
Q3THS6	Mat2a	> 2.0
Q8K310	Matr3	< 2.0
Q99MR8	Mccc1	< 2.0
Q3ULD5	Mccc2	< 2.0
P54276	Msh6	> 2.0
Q7TPV4	Mybbp1a	> 2.0
Q61879	Myh10	< 2.0
Q8VDD5	Myh9	< 2.0
Q8BWZ3	Naa25	> 2.0
Q8BP47	Nars	< 2.0
P09405	Ncl	> 2.0
P10493	Nid1	< 2.0
Q9D6Z1	Nop56	> 2.0
Q6DFW4	Nop58	> 2.0
Q61937	Npm1	> 2.0
P09103	P4hb	< 2.0
P29341	Pabpc1	> 2.0
Q9DCL9	Paics	> 2.0
Q99LX0	Park7	> 2.0
P11103	Parp1	> 2.0
P24610	Pax3	< 2.0
P47239	Pax7	< 2.0
P60335	Pcbp1	> 2.0
Q91ZA3	Pcca	< 2.0
Q99MN9	Pccb	< 2.0
P17918	Pcna	> 2.0
Q05920	Pcx	< 2.0
P35486	Pdha1	< 2.0
Q8QZR7	Pdik1l	< 2.0
P70296	Pebp1	< 2.0
Q9DBJ1	Pgam1	> 2.0
O70250	Pgam2	> 2.0
P09411	Pgk1	< 2.0
P09041	Pgk2	< 2.0
O35129	Phb2	> 2.0
Q8R1K4	Phykpl	NA
P53657	Pklr	< 2.0
P52480	Pkm	NA
P17742	Ppia	> 2.0
Q9CR16	Ppid	> 2.0
Q99KR7	Ppif	> 2.0
Q9D868	Ppih	> 2.0
P35700	Prdx1	> 2.0
Q9JIF0	Prmt1	> 2.0
Q99JF8	Psip1	> 2.0
P62334	Psmc6	< 2.0
Q8VDM4	Psmd2	< 2.0
P17225	Ptbp1	> 2.0
Q8BHD7	Ptbp3	NA
Q9R0Q7	Ptges3	> 2.0
Q3UEB3	Puf60	< 2.0
Q922W5	Pycr1	< 2.0
Q922Q4	Pycr2	> 2.0
P61027	Rab10	< 2.0
Q9DD03	Rab13	< 2.0
Q8K386	Rab15	< 2.0
P62821	Rab1a	NA
Q9D1G1	Rab1b	< 2.0
Q6PHN9	Rab35	< 2.0
P55258	Rab8a	< 2.0
P61028	Rab8b	< 2.0
Q64012	Raly	< 2.0
P62827	Ran	> 2.0

Accession	Gene Symb	ESC/MEF
Q61820	Rasl2-9	NA
Q60972	Rbbp4	> 2.0
Q60973	Rbbp7	< 2.0
O89086	Rbm3	< 2.0
Q8VH51	Rbm39	< 2.0
Q9WV02	Rbmx	< 2.0
Q91VM5	Rbmxl1	NA
Q9WUK4	Rfc2	> 2.0
Q99J62	Rfc4	> 2.0
P56716	Rp1	< 2.0
Q6ZWV3	Rpl10	< 2.0
P53026	Rpl10a	< 2.0
P86048	Rpl10l	< 2.0
Q9CXW4	Rpl11	< 2.0
P35979	Rpl12	< 2.0
P47963	Rpl13	< 2.0
Q9CR57	Rpl14	> 2.0
Q9CZM2	Rpl15	< 2.0
P62717	Rpl18a	> 2.0
P84099	Rpl19	< 2.0
P67984	Rpl22	< 2.0
P62751	Rpl23a	> 2.0
P61358	Rpl27	> 2.0
P14115	Rpl27a	< 2.0
P62911	Rpl32	< 2.0
P17932	Rpl32-ps	NA
P83882	Rpl36a	< 2.0
Q9D8E6	Rpl4	> 2.0
P47962	Rpl5	> 2.0
P47911	Rpl6	> 2.0
P14148	Rpl7	< 2.0
P12970	Rpl7a	> 2.0
P63325	Rps10	< 2.0
P62281	Rps11	< 2.0
P63323	Rps12	< 2.0
P62301	Rps13	< 2.0
P62264	Rps14	< 2.0
P62245	Rps15a	< 2.0
P14131	Rps16	< 2.0
P62270	Rps18	< 2.0
P25444	Rps2	< 2.0
P62267	Rps23	< 2.0
P62852	Rps25	< 2.0
P62855	Rps26	< 2.0
P62983	Rps27a	< 2.0
P62908	Rps3	< 2.0
P97351	Rps3a1	NA
P62702	Rps4x	< 2.0
P62754	Rps6	< 2.0
P62242	Rps8	< 2.0
Q6ZWN5	Rps9	< 2.0
Q9WTM5	Ruvbl2	> 2.0
Q9D1J3	Sarnp	< 2.0
Q8K2B3	Sdha	< 2.0
P19324	Serpinh1	< 2.0
Q99NB9	Sf3b1	< 2.0
Q921M3	Sf3b3	> 2.0
Q62407	Speg	< 2.0
Q62261	Sptbn1	NA
Q6PDM2	Srsf1	> 2.0
P84104	Srsf3	> 2.0
Q8VE97	Srsf4	> 2.0
O35326	Srsf5	< 2.0
Q3TWW8	Srsf6	> 2.0
Q8BL97	Srsf7	> 2.0
Q80ZW0	Stk35	> 2.0
Q69ZM6	Stk36	< 2.0
Q9WUM5	Suclg1	< 2.0
Q920B9	Supt16	NA
Q7TMK9	Syncrip	< 2.0
Q9WVA4	Tagln2	< 2.0
Q8C4J7	Tbl3	> 2.0
P10711	Tcea1	NA
Q9QVN7	Tcea2	< 2.0
O08784	Tcof1	> 2.0
Q8K3F7	Tdh	< 2.0
Q569Z6	Thrap3	> 2.0
P40142	Tkt	> 2.0
Q62318	Trim28	> 2.0
Q923J1	Trpm7	< 2.0

Accession	Gene Symb	ESC/MEF
Q9WTR1	Trpv2	< 2.0
P68369	Tuba1a	< 2.0
P05213	Tuba1b	> 2.0
P68373	Tuba1c	< 2.0
P68368	Tuba4a	> 2.0
A2AQ07	Tubb1	< 2.0
Q7TMM9	Tubb2a	< 2.0
Q9CWF2	Tubb2b	< 2.0
Q9ERD7	Tubb3	< 2.0
Q9D6F9	Tubb4a	NA
P68372	Tubb4b	NA
P99024	Tubb5	< 2.0
Q922F4	Tubb6	< 2.0
Q8BFR5	Tufm	< 2.0
P62984	Uba52	< 2.0
P0CG49	Ubb	> 2.0
P0CG50	Ubc	< 2.0
Q6WKZ8	Ubr2	< 2.0
P52624	Upp1	> 2.0
Q8CGR7	Upp2	< 2.0
Q01853	Vcp	< 2.0
P62960	Ybx1	> 2.0
P62259	Ywhae	> 2.0

NA: Data of RNA-Seq is not available

Table S2. Function and ontologies of selected proteins

Gene symbol	Function	GO Term Finder							MGI GO
		RNA processing	ribonucleoprotein complex biogenesis	RNA splicing	mRNA processing	gene expression	RNA splicing, via transesterification reactions	RNA splicing, via transesterification reactions with bulged adenosine as nucleophile	DNA binding OR DNA-binding transcription factor binding
Acacb	acetyl-Coenzyme A carboxylase					Acacb			
Acin1	apoptotic chromatin condensation inducer 1	Acin1		Acin1	Acin1	Acin1	Acin1	Acin1	YES
Bclaf1	BCL2-associated transcription factor 1					Bclaf1			YES
Cdc5l	cell division cycle 5-like	Cdc5l		Cdc5l	Cdc5l	Cdc5l	Cdc5l	Cdc5l	
Dazap1	DAZ associated protein 1	Dazap1		Dazap1	Dazap1	Dazap1	Dazap1	Dazap1	
Ddx21	DEAD box helicase 21	Ddx21	Ddx21			Ddx21			
Ddx39	DEAD box helicase 39	Ddx39		Ddx39	Ddx39	Ddx39	Ddx39	Ddx39	
Ddx39b	DEAD box helicase 39b	Ddx39b	Ddx39b	Ddx39b	Ddx39b	Ddx39b	Ddx39b	Ddx39b	
Ddx3x	DEAD box helicase 3	Ddx3x	Ddx3x			Ddx3x			
Eef1a1	eukaryotic translation elongation factor 1 alpha 1					Eef1a1			
Eftud2	elongation factor Tu GTP binding domain containing 2	Eftud2		Eftud2	Eftud2	Eftud2	Eftud2	Eftud2	
Eif5a	eukaryotic translation initiation factor 5A					Eif5a			
Eno1	enolase 1, alpha					Eno1			YES
Fbl	fibrillarin	Fbl	Fbl			Fbl			
Fubp1	far upstream element binding protein 1					Fubp1			YES
G3bp1	GTPase activating protein binding protein 1					G3bp1			YES
Gapdh	glyceraldehyde-3-phosphate dehydrogenase					Gapdh			
H2afz	H2A.Z variant histone 1					H2afz			YES
Helb	helicase B	Helb	Helb			Helb			
Hist1h3g	H3 clustered histone 8					Hist1h3g			
Hmgb2	high mobility group box 2					Hmgb2			YES
Hnrnpab	heterogeneous nuclear ribonucleoprotein A/B					Hnrnpab			YES
Hnrnpc	heterogeneous nuclear ribonucleoprotein C	Hnrnpc		Hnrnpc	Hnrnpc	Hnrnpc	Hnrnpl	Hnrnpl	
Hnrnpl	heterogeneous nuclear ribonucleoprotein L	Hnrnpl		Hnrnpl	Hnrnpl	Hnrnpl	Hnrnpm	Hnrnpm	
Hnrnpm	heterogeneous nuclear ribonucleoprotein M	Hnrnpm		Hnrnpm	Hnrnpm	Hnrnpm	Hnrnpu	Hnrnpu	
Hnrnpu	heterogeneous nuclear ribonucleoprotein U	Hnrnpu		Hnrnpu	Hnrnpu	Hnrnpu			YES
Hsp90aa1	heat shock protein 90, alpha, class A member 1		Hsp90aa1			Hsp90aa1			
Hsp90ab1	heat shock protein 90, alpha, class B member 1		Hsp90ab1			Hsp90ab1			
Hspa8	heat shock protein 8	Hspa8		Hspa8	Hspa8	Hspa8	Hspa8	Hspa8	
Hspb1	heat shock protein 1					Hspb1			
Hspd1	heat shock protein 1 (chaperonin)					Hspd1			
Mak16	MAK16 homolog	Mak16	Mak16			Mak16			
Msh6	mutS homolog 6					Msh6			YES
Mybbp1a	MYB binding protein 1a		Mybbp1a			Mybbp1a			YES
Naa25	N(alpha)-acetyltransferase 25					Naa25			
Ncl	nucleolin	Ncl	Ncl	Ncl	Ncl	Ncl	Ncl	Ncl	YES
Nop56	NOP56 ribonucleoprotein		Nop56						
Nop58	NOP58 ribonucleoprotein		Nop58						
Npm1	nucleophosmin 1	Npm1	Npm1	Npm1	Npm1	Npm1	Npm1	Npm1	YES
Pabpc1	poly(A) binding protein, cytoplasmic 1	Pabpc1		Pabpc1	Pabpc1	Pabpc1			
Park7	Parkinson disease 7					Park7			YES
Parp1	poly (ADP-ribose) polymerase family, member 1					Parp1			YES
Pcbp1	poly(rC) binding protein 1	Pcbp1			Pcbp1	Pcbp1			YES
Pcna	proliferating cell nuclear antigen					Pcna			YES
Phb2	prohibitin 2					Phb2			
Ppia	peptidylprolyl isomerase A					Ppia			
Ppid	peptidylprolyl isomerase D					Ppid			
Prmt1	protein arginine N-methyltransferase 1	Prmt1		Prmt1		Prmt1			
Psip1	PC4 and SFRS1 interacting protein 1	Psip1	Psip1	Psip1	Psip1	Psip1	Psip1	Psip1	YES
Ptbp1	polypyrimidine tract binding protein 1	Ptbp1		Ptbp1	Ptbp1	Ptbp1	Ptbp1	Ptbp1	YES
Ptges3	prostaglandin E synthase 3		Ptges3			Ptges3			
Rbbp4	retinoblastoma binding protein 4					Rbbp4			
Ran	RAN, member RAS oncogene family		Ran						
Rpl14	ribosomal protein L14	Rpl14	Rpl14			Rpl14			
Rpl18a	ribosomal protein L18A					Rpl18a			
Rpl23a	ribosomal protein L23A		Rpl23a			Rpl23a			
Rpl27	ribosomal protein L27	Rpl27	Rpl27			Rpl27			
Rpl4	ribosomal protein L4					Rpl4			
Rpl5	ribosomal protein L5	Rpl5	Rpl5			Rpl5			
Rpl6	ribosomal protein L6		Rpl6			Rpl6			
Rpl7a	ribosomal protein L7A	Rpl7a	Rpl7a			Rpl7a			
Ruvbl2	RuvB-like protein 2		Ruvbl2			Ruvbl2			
Sf3b3	splicing factor 3b, subunit 3	Sf3b3		Sf3b3	Sf3b3	Sf3b3	Sf3b3	Sf3b3	
Srsf1	serine and arginine-rich splicing factor 1	Srsf1	Srsf1	Srsf1	Srsf1	Srsf1	Srsf1	Srsf1	
Srsf3	serine and arginine-rich splicing factor 3	Srsf3		Srsf3	Srsf3	Srsf3	Srsf3	Srsf3	
Srsf4	serine and arginine-rich splicing factor 4	Srsf4		Srsf4	Srsf4	Srsf4	Srsf4	Srsf4	
Srsf6	serine and arginine-rich splicing factor 6	Srsf6	Srsf6	Srsf6	Srsf6	Srsf6	Srsf6	Srsf6	
Srsf7	serine and arginine-rich splicing factor 7	Srsf7		Srsf7	Srsf7	Srsf7	Srsf7	Srsf7	
Tbl3	transducin (beta)-like 3	Tbl3	Tbl3			Tbl3			
Tcof1	treacle ribosome biogenesis factor 1					Tcof1			
Thrap3	thyroid hormone receptor associated protein 3	Thrap3		Thrap3	Thrap3	Thrap3	Thrap3	Thrap3	YES
Trim28	tripartite motif-containing 28					Trim28			YES
Ybx1	Y box protein 1	Ybx1		Ybx1	Ybx1	Ybx1			YES

Table S3. Oligonucleotide sequences for plasmid construction

Guide RNA Sequences

sgNanog-1	5'-CACC GCTGTAAGGTGACCCAGACT-3'
	5'-AAAC AGTCTGGGTCACCTTACAGC-3'
sgNanog-2	5'-CACC GTGGGGCGTGGGTGCCGCCT-3'
	5'-AAAC AGGCGGCACCCACGCCCCAC-3'
sgNanog-3	5'-CACC GCTCAAGGCGATAGATTTAA-3'
	5'-AAAC TTAAATCTATCGCCTTGAGC-3'
Mock (sgGal4)	5'-CACC GAACGACTAGTTAGGCGTGTA-3'
	5'-AAAC TACACGCCTAACTAGTCGTTC-3'
sgRex1(+)	5'-CACC GCAAAGACAAGTGGCCAGAA-3'
	5'-AAAC TTCTGGCCACTTGTCTTTGC-3'
sgRex1(-)	5'-CACC GCTCTTCCGCCCGGCCCTTTC-3'
	5'-AAAC GAAAGGGCCGGGCGGAAGAGC-3'

shRNA Oligo Sequences

shBclaf1-1	5'-GATCCGGATCTGGCTCTGTTGGAAATTTCAAGAGAATTTCCAACAGAGCCAGATCCTTTTTTGGAAAG-3'
	5'-AATTCTTTCCAAAAAAGGATCTGGCTCTGTTGGAAATTCTCTTGAAATTTCCAACAGAGCCAGATCCG-3'
shBclaf1-2	5'-GATCCGGTTCATCATGTGAAAGAATTCAAGAGATTCTTTCACATGATGAACCTTTTTTGGAAAG-3'
	5'-AATTCTTTCCAAAAAAGGTTCATCATGTGAAAGAATCTCTTGAATTCTTTCACATGATGAACCG-3'
shFubp1-1	5'-GATCCGCTGGTACATCATTGAATTCATTCAAGAGATGAATTCAATGATGTACCAGCTTTTTTGGAAAG-3'
	5'-AATTCTTTCCAAAAAAGCTGGTACATCATTGAATTCATCTCTTGAATGAATTCAATGATGTACCAGCG-3'
shFubp1-2	5'-GATCCGGACAGGTTGATTATACAATTCAAGAGATTGTATAATCAACCTGTCCTTTTTTGGAAAG-3'
	5'-AATTCTTTCCAAAAAAGGACAGGTTGATTATACAATCTCTTGAATTGTATAATCAACCTGTCCG-3'
shMsh6-1	5'-GATCCGAGGTGCATGAGGCTTATTTATTCAAGAGATAAATAAGCCTCATGCACCTCTTTTTTGGAAAG-3'
	5'-AATTCTTTCCAAAAAAGAGGTGCATGAGGCTTATTTATCTCTTGAATAAATAAGCCTCATGCACCTCG-3'
shMsh6-2	5'-GATCCGCTACTGAGTAAGATTCATTTCAAGAGAATGAATCTTACTCAGTAGCTTTTTTGGAAAG-3'
	5'-AATTCTTTCCAAAAAAGCTACTGAGTAAGATTCATTCTCTTGAAATGAATCTTACTCAGTAGCG-3'
shPark7-1	5'-GATCCGCAGTGTAGCCGTGATGTAATTTCAAGAGAATTACATCACGGCTACACTGCTTTTTTGGAAAG-3'
	5'-AATTCTTTCCAAAAAAGCAGTGTAGCCGTGATGTAATTCTCTTGAAATTACATCACGGCTACACTGCG-3'
shPark7-2	5'-GATCCGCTGCAGTCTTTAAGAAATTTCAAGAGAATTTCTTAAAGACTGCAGCTTTTTTGGAAAG-3'
	5'-AATTCTTTCCAAAAAAGCTGCAGTCTTTAAGAAATTCTCTTGAAATTTCTTAAAGACTGCAGCG-3'
shPsip1-1	5'-GATCCGGTTATTGATGAAGATAAATAACCTGACCCATTATTTATCTTCATCAATAACCTTTTTTGGAAAG-3'
	5'-AATTCTTTCCAAAAAAGGTTATTGATGAAGATAAATAATGGGTCAGGTTATTTATCTTCATCAATAACCG-3'
shPsip1-2	5'-GATCCGCAATGAGGATGTGACTAAAGTTCAAGAGACTTTAGTCACATCCTCATTGCTTTTTTGGAAAG-3'
	5'-AATTCTTTCCAAAAAAGCAATGAGGATGTGACTAAAGTCTCTTGAACTTTAGTCACATCCTCATTGCG-3'
shThrap3-1	5'-GATCCGGGATATATAGTAATATTATAACCTGACCCATTATAATATTACTATATATCCCTTTTTTGGAAAG-3'
	5'-AATTCTTTCCAAAAAAGGGATATATAGTAATATTATAATGGGTCAGGTTATAATATTACTATATATCCCG-3'
shThrap3-2	5'-GATCCGGAGCTGGATGAGCACGATAATTCAAGAGATTATCGTGCTCATCCAGCTCCTTTTTTGGAAAG-3'
	5'-AATTCTTTCCAAAAAAGGAGCTGGATGAGCACGATAATCTCTTGAATTATCGTGCTCATCCAGCTCCG-3'
shOct4	5'-GATCCGCCTTAAGAACATGTGTAATTCAAGAGATTACACATGTTCTTAAGGCTTTTTTGGAAAG-3'
	5'-AATTCTTTCCAAAAAAGCCTTAAGAACATGTGTAATCTCTTGAATTACACATGTTCTTAAGGCG-3'

Donor Oligo Sequences (Oligo's for Amplying *Nanog* promoter region)

Nanog (Promoter)	5'-TAGCCTGCAGGTCATCCCCCACTTGACCTGAAAC-3'
	5'-TATCGAATTCGTCCAGCCTTCCCACAGAAAGAGC-3'

Table S4. Primer sequences for PCR

***Nanog* enrichment primers (For CAPTURE & ChIP-qPCR)**

CAPTURE	5'-GGTCACCTTACAGCTTCTTTTG-3'
	5'-TATTCTCCCAGGCACCCAG-3'
ChIP-qPCR	5'-GCAGGACCTACCCTTTAAATC-3'
	5'-CCCACAGAAAGAGCAAGAC-3'

RT-qPCR primers

mThrap3	5'-AGAAAAGCCCAGAGATACACAG-3'
	5'-TATTTTCCCTCAGCCTTGTAGCC-3'
mBclaf1	5'-TGTTGAAGATGACGAAGAGACC-3'
	5'-CAAGTTCTGCTCCCTGTTGC-3'
mPsip1	5'-CGAGGAAGAAAGAGAAAGGCTG-3'
	5'-AGGTCTGCCTCTTGGTTTGG-3'
mPark7	5'-TGCAGTGTAGCCGTGATGTA-3'
	5'-TATGGTCCCTGCGTTTTTGC-3'
mFubp1	5'-GCTACAACCCAAACGAACGG-3'
	5'-AGGAACTGCTTGACCCATTTTC-3'
mMsh6	5'-CGTGCCTCCCAGTTCTTGTG-3'
	5'-GGACAGATTTCCCTTTCTTCCG-3'
mTBP	5'-TATCTGCTGGCGGTTTGGC-3'
	5'-TGAAATAGTGATGCTGGGCAC-3'
mT	5'-GCTTCAAGGAGCTAACTAACGAG-3'
	5'-CCAGCAAGAAAGAGTACATGGC-3'
mNanog	5'-ACCTGAGCTATAAGCAGGTTAAGAC-3'
	5'-GTGCTGAGCCCTTCTGAATCAGAC-3'
mRex1	5'-ATACCACTGACCAAAAAGCAGG-3'
	5'-GCCACTTGTCTTTGCCGTTTTC-3'
mOct4	5'-AGCATTGAGAACCGTGTGAGG-3'
	5'-AACCATACTCGAACCACATCC-3'
mFgf5	5'-AAGTAGCGCGACGTTTTCTTC-3'
	5'-CTGGAAACTGCTATGTTCCGAG-3'
mMeox1	5'-ACAGCCTTCACCAAGGAGC-3'
	5'-CCCCTTCACACGTTTCCA-3'
mGata6	5'-GGCTCTGTCCCTATGACTCC-3'
	5'-TGATGCCCCTACCCCTGAG-3'

Genotyping primers

WT	5'-AAACGGCAAAGACAAGTGGC-3'
	5'-ACTGCCAAAGTTGGCCATTC-3'
Knock-IN (EB5/ReKO-Puro)	5'-TGCAACTTACCTCCGGGATG-3'
	5'-ACTGCCAAAGTTGGCCATTC-3'
Cre (EB5/ReKO)	5'-CCGACGCCGACCAACACC-3'
	5'-ACTGCCAAAGTTGGCCATTC-3'
